# Automated Gleason Scoring and Tumor Quantification in Prostate Core Needle Biopsy Images Using Deep Neural Networks and Its Comparison with Pathologist-Based Assessment

**DOI:** 10.3390/cancers11121860

**Published:** 2019-11-25

**Authors:** Han Suk Ryu, Min-Sun Jin, Jeong Hwan Park, Sanghun Lee, Joonyoung Cho, Sangjun Oh, Tae-Yeong Kwak, Junwoo Isaac Woo, Yechan Mun, Sun Woo Kim, Soohyun Hwang, Su-Jin Shin, Hyeyoon Chang

**Affiliations:** 1 Department of Pathology, Seoul National University College of Medicine, Seoul 03080, Korea; karlnash@naver.com (H.S.R.); hopemd@hanmail.net (J.H.P.); 2 Department of Pathology, Seoul National University Hospital, Seoul 03080, Korea; 3 Department of Pathology, Bucheon St. Mary’s Hospital, College of Medicine, The Catholic University of Korea, Bucheon, Gyeonggi-do 14647, Korea; seasy@hanmail.net; 4 Department of Pathology, SMG-SNU Boramae Medical Center, Seoul 07061, Korea; 5 Deep Bio Inc., 1201 HanWha BizMetro, 242, Digital-ro, Guro-gu, Seoul 08394, Korea; deep.shlee@gmail.com (S.L.); jycho@deepbio.co.kr (J.C.); me@juneoh.net (S.O.); tykwak@deepbio.co.kr (T.-Y.K.); jwwoo@deepbio.co.kr (J.I.W.); ycmun@deepbio.co.kr (Y.M.); swkim@deepbio.co.kr (S.W.K.); 6 Department of Pathology and Translational Genomics, Samsung Medical Center, Sungkyunkwan University School of Medicine, Seoul 06351, Korea; juzzsaw@gmail.com; 7 Department of Pathology, Gangnam Severance Hospital, Yonsei University College of Medicine, Seoul 06273, Korea

**Keywords:** gleason scoring system, deep neural network, prostate cancer, prostate core needle biopsy

## Abstract

The Gleason grading system, currently the most powerful prognostic predictor of prostate cancer, is based solely on the tumor’s histological architecture and has high inter-observer variability. We propose an automated Gleason scoring system based on deep neural networks for diagnosis of prostate core needle biopsy samples. To verify its efficacy, the system was trained using 1133 cases of prostate core needle biopsy samples and validated on 700 cases. Further, system-based diagnosis results were compared with reference standards derived from three certified pathologists. In addition, the system’s ability to quantify cancer in terms of tumor length was also evaluated via comparison with pathologist-based measurements. The results showed a substantial diagnostic concordance between the system-grade group classification and the reference standard (0.907 quadratic-weighted Cohen’s kappa coefficient). The system tumor length measurements were also notably closer to the reference standard (correlation coefficient, *R* = 0.97) than the original hospital diagnoses (*R* = 0.90). We expect this system to assist pathologists to reduce the probability of over- or under-diagnosis by providing pathologist-level second opinions on the Gleason score when diagnosing prostate biopsy, and to support research on prostate cancer treatment and prognosis by providing reproducible diagnosis based on the consistent standards.

## 1. Introduction

Prostate cancer is a leading type of cancer in men worldwide. In 2017, 161,360 new cases of prostate cancer were reported in the USA, with an estimated 26,730 deaths [1]. Core needle biopsy is the main method for diagnosis of prostate cancer and recommended to patients whose results from initial screening tests (e.g., a prostate-specific antigen test, and digital rectal exam) are suspicious of prostate cancer [2].

The management of prostate cancer differs depending on the patients’ life expectancy, prostate-specific antigen level, and Gleason score.

The Gleason score is a grading system widely used for evaluating prostate cancer tissues and is the standard for prognosis prediction and treatment selection. It was first developed by Donald Gleason and the Veterans’ Administration Cooperative Urologic Research Group between 1966 and 1974. The Gleason score is based on histologic architecture of tumor and underwent significant revisions in 2005 and 2014 [3,4,5,6]. However, as the score is qualitative and subjective, it considerably varies with respect to inter- and intra-observer variability among pathologists [7,8].

Therefore, a new five-group grading system for prostate cancer was proposed in 2013 at Johns Hopkins Hospital; specifically, prognostic grade group 1 (Gleason score ≤ 6), prognostic grade group 2 (Gleason score 3 + 4 = 7), prognostic grade group 3 (Gleason score 4 + 3 = 7), prognostic grade group 4 (Gleason score 8), and prognostic grade group 5 (Gleason scores 9 and 10) [9]. These groups were validated in a multi-institutional study, including Johns Hopkins Hospital, the Memorial Sloan-Kettering Cancer Center, University of Pittsburgh, Cleveland Clinic, and Karolinska Institute based on patients’ prognosis and biochemical recurrence [10]. Moreover, at the ISUP 2014 consensus conference, the use of a new prostate cancer grading system in conjunction with the Gleason system was accepted by a broad consensus (90%) [4].

The development of the whole-slide imaging system, its approval by the Food and Drug Administration, and studies proving its feasibility for primary diagnosis led to increasing use and acceptance of digital images for surgical pathology [11]. The digitization of pathological images has also enabled computational pathology and facilitated research on automated analyses using approaches such as machine learning.

Among various machine learning techniques, deep learning has recently been gaining attention for use in various fields. Supported by modern hardware and big data, deep learning utilizes deep artificial neural networks to automatically learn complex high-level representations, thus avoiding hand-crafted features. Based on studies on other medical imaging modalities, applications of deep learning to pathological images have been studied [12,13].

Recently, many studies have been conducted to apply deep learning to whole slide images (WSIs) diagnosis in several types of cancers, including breast, brain, colon and lung cancer [14,15,16,17,18,19,20,21]. In prostate cancer, several attempts have been made to detect prostate cancer [22,23] and Gleason scoring [24,25,26] using tissue microarrays (TMA), prostatectomy and core needle biopsy samples.

In this paper, we propose an automated Gleason scoring system for prostate core needle biopsy samples: DeepDx Prostate. To assist pathologists toward more precise and consistent diagnoses, DeepDx Prostate features a deep neural network architecture trained on more than 1000 annotated WSIs, along with a viewer and a set of manual annotation tools. We report its performance compared to the results of a multi-institutional reference standard established by certified pathologists and its ability to quantify cancer in terms of tumor length compared with pathologist-based measurements.

## 2. Results

**Figure 1:**
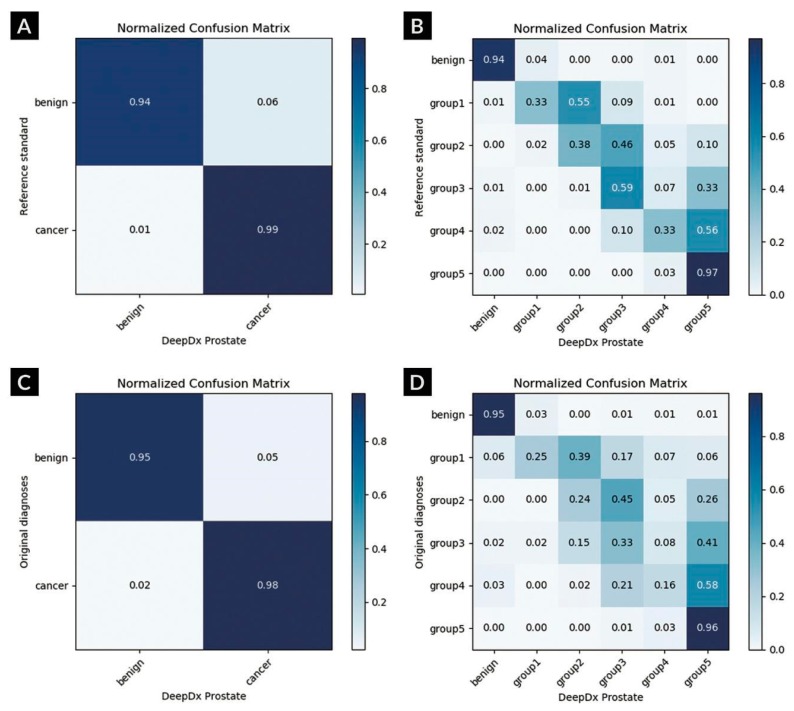
Normalized confusion matrices between DeepDx Prostate and diagnoses. (**A**) Binary and (**B**) categorical results against reference standard. (**C**) Binary and (**D**) categorical results against original hospital diagnoses.

**Figure 2:**
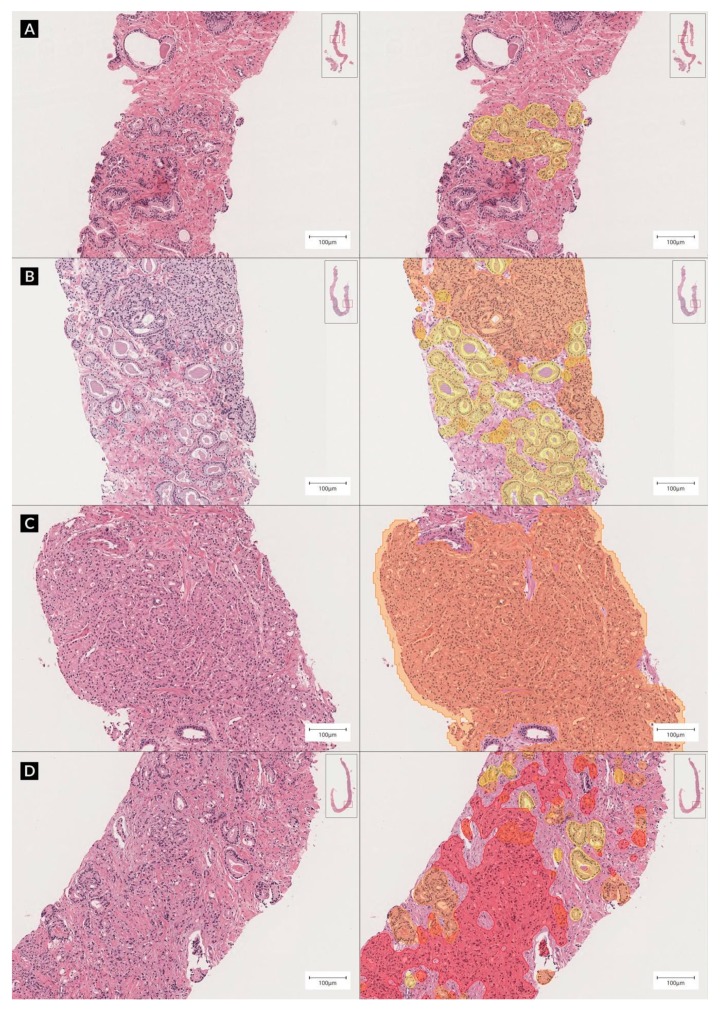
Representative examples of core biopsy slides, hematoxylin and eosin (H&E) staining (left), and DeepDx Prostate analysis (right) at ×10 magnification. Core needle biopsy of prostate corresponding to grade groups (**A**) 1, (**B**) 2, (**C**) 4, and (**D**) 5. Highlights in yellow, orange, and red correspond to regions of Gleason patterns 3, 4, and 5, respectively.

The proposed system performance according to grade groups showed that the system achieved a higher diagnostic concordance to the reference standard (0.907 in quadratic-weighted kappa) compared to the original hospital diagnoses (0.870) in the validation dataset. The quadratic-weighted kappa score between individual pathologists ranged from 0.896 to 0.920 (mean, 0.907), and the mean between the system score and that of each pathologist was 0.899. The detailed results are listed in Table 1. The normalized confusion matrices and representative examples of the analysis through DeepDx Prostate are shown in Figure 1 and Figure 2, respectively.

**Figure 3:**
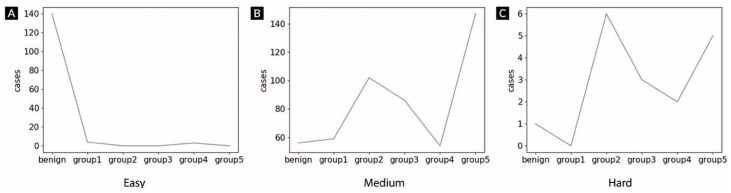
Distributions of grade groups according to diagnostic difficulty: (**A**) easy, (**B**) medium, and (**C**) hard. Grade groups were obtained from the reference standard.

**Figure 4:**
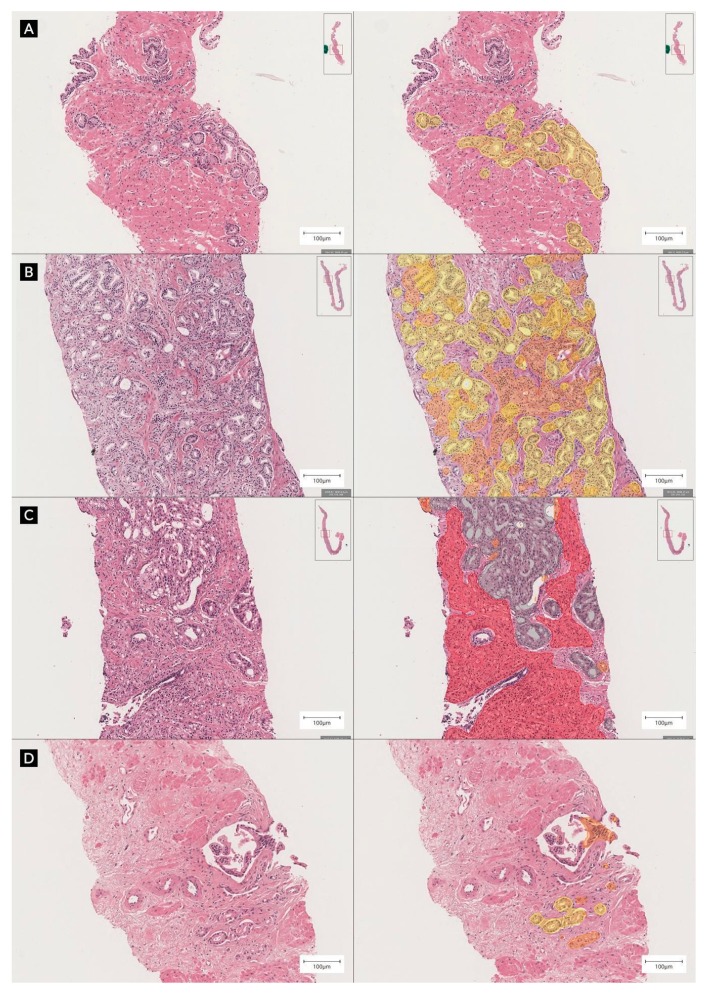
Representative examples of each level of difficulty, H&E staining (left), and DeepDx Prostate analysis (right) at ×10 magnification. (**A**) Easy-level image showing well-formed individual glands in grade group 1. (**B**) Medium level image showing well-formed individual glands intermingled with fused glands. (**C**) Hard-level image showing high-grade tumor intermingled with intraductal carcinoma of the prostate, and (**D**) very small foci of suspicious tumorous lesion. Highlights in yellow, orange, and red correspond to regions of Gleason patterns 3, 4, and 5, respectively.

By excluding cases with ambiguous or empty responses from pathologists, 674 from the 700 cases were available for difficulty stratification: 147 easy, 505 medium, and 22 hard. Among the easy cases, 140 were benign and the system’s analysis showed the highest diagnostic concordance with the reference standards and original hospital diagnoses. In contrast, the hard cases showed the worst correlation, and the slides with medium difficulty showed moderate correlation. The corresponding kappa coefficient matrices are detailed in Table 2. Figure 3 shows the distribution within each difficulty level, and Figure 4 shows the representative images of each level. The concordance between pathologists was also measured at each difficulty level, as listed in Table 3. Similar to the DeepDx Prostate results, the correlation between the pathologist-based scores was lower for the hard level than for the easy level.

**Figure 5:**
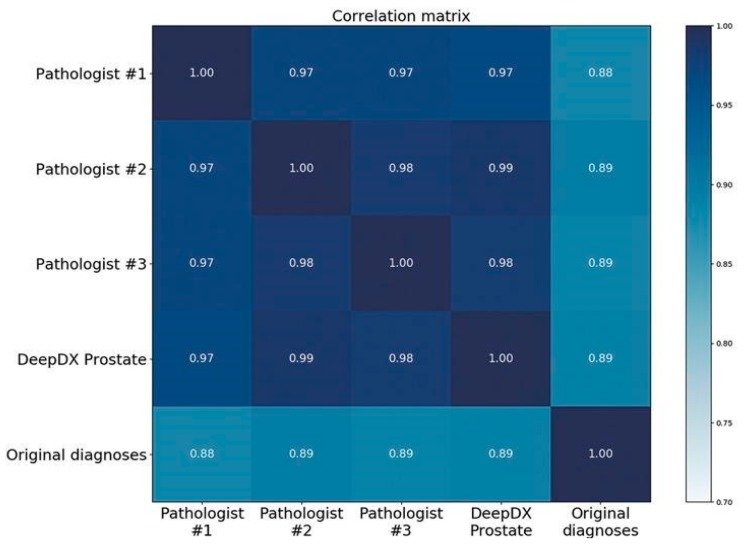
Correlation matrix of tumor lengths.

The DeepDx Prostate results of tumor length measurements were also notably closer to the reference standard than the original hospital diagnoses. Correlation coefficient *R* between the tumor lengths of the pathologists and DeepDx Prostate was above 0.97, whereas the correlations for the original diagnoses were all below 0.90, as shown in the correlation matrix of Figure 5.

There were 76 cases within the validation set for which one or more pathologists reported poor image quality. Among them, 49 cases showed faults during slide preparation and 33 cases showed errors during scanning. However, none of these cases impeded the diagnoses through DeepDx Prostate or by the pathologists (Figure S1 shows poor quality images).

Among the 11 atypical small acinar proliferation (ASAP) cases, one or more pathologists marked six cases as requiring immunohistochemical staining, such as high molecular weight cytokeratin (HMW-CK; Table S1 shows details of ASAP cases). Five of these cases were confirmed to exhibit acinar adenocarcinoma or atypical small acinar proliferation (ASAP) under HMW-CK staining (Figure S2).

## 3. Discussion

In this study, we introduced an automated Gleason scoring system, trained it using 1133 cases of prostate core needle biopsy samples, and validated it on 700 cases. The system diagnoses were compared with the reference standard derived from three certified pathologists. The results showed a substantial diagnostic concordance between the system-grade group classification and reference standard (non-weighted Cohen’s kappa coefficient was 0.615); this value was higher than that of the original hospital diagnoses (0.524), or the previously reported agreements between pathologists, ranging between 0.4 and 0.6 [7,8]. As the Gleason score provides an important index of prognosis and is one of the key determinants for selecting treatment modalities, the variability between the diagnoses of different pathologists is of clinical concern. The automated Gleason scoring system can help pathologists by providing diagnoses based on consistent standards and clear percentages of each Gleason pattern; this could help urologists in selecting appropriate treatment modalities.

Several studies have addressed automated Gleason scoring by using deep learning. Arvaniti et al. [24] reported a deep learning system that achieved comparable results with pathologist evaluations on Gleason scoring with respect to resection specimen of prostate tissue microarray. Likewise, Nagpal et al. used deep learning based on a modified InceptionV3 classifier to obtain reliable performance in the grade-group determination on resection specimens from the TCGA dataset [25].

Both studies focused on patch-level neural networks, whereas our automated Gleason system implements a pixel-level segmentation neural network, possibly achieving more reliable Cohen’s kappa coefficient (0.615) and Cohen’s quadratic kappa coefficient (0.907). Similarly, Campanella et al. [22] trained a patch-level neural network with a massive dataset containing over 10,000 WSIs to access slide-level prediction and determine whether slides contain cancerous regions through multiple instance learning. They obtained a high area under the receiver operating characteristic curve (0.98) without requiring region-level annotations from pathologists.

Moreover, Arvaniti et al. [24] and Nagpal et al. [25] showed the possibility of better risk stratification in biochemical recurrence or disease progression through deep learning. Nagpal et al. [25] suggested a fine-grained Gleason scoring system with interpolation methods to enhance biomarkers in prognostic factors. Our measurements, including grade groups and tumor length obtained from the automated Gleason scoring system will be studied in survival analysis to test whether these factors are significant and remain consistent.

We asked three pathologists to assess not only the Gleason score but also the difficulty level (easy, medium, or hard) of each case. Concordance analysis with respect to difficulty level revealed that diagnostic concordance was highest in easy cases and deteriorated as case difficulty increased, and this trend was consistent with the correlation analysis between pathologists. Benign cases composed the majority of easy cases, whereas notable peaks were observed for grade groups 2 and 5 at both the medium and hard levels. In grade group 2 (Gleason score 3 + 4), variable judgment about the composition ratios of Gleason patterns 3 and 4 could be the reason for the lower concordance rate. While reviewing the hard cases of grade group 5, we found several cases with lesions suspicious of intraductal carcinoma of the prostate (IDC-P), requiring differential diagnosis from cribriform Gleason pattern 4 through additional immunohistochemical staining.

We additionally considered cancer quantification in each case. Several methods have been used in previous studies to evaluate the extent of tumor. Evaluation metrics include the number/fraction of positive cores, tumor percentage or linear millimeters of carcinoma per core, linear percentage carcinoma or linear millimeters of carcinoma in core with the greatest tumor volume, and total linear millimeters of carcinoma across all cores [27,28]. There is no consensus regarding which method is the best in measuring the extent of tumor in prostate core needle biopsy; several studies have demonstrated relationships between their respective methods of tumor measurement and certain prognostic factors. For instance, Quintal et al. [29] found that the percentage of total carcinoma length (in millimeters) in all cores of a needle biopsy was the strongest predictor for stages beyond pT2 and the risk of biochemical recurrence following radical prostatectomy. Brimo et al. [30] found that the fraction of positive cores, the total percentage of carcinoma, and both the total and greatest cancer core length were closely related to the pathologic stage and biochemical failure.

In this study, we asked pathologists to measure carcinoma in terms of linear millimeters. Tumor lengths measured by pathologists from WSIs and those automatically determined by the system showed a high correlation, whereas a notable difference was observed in the measurements obtained from glass slides of the source hospitals. To determine the tumor area, pathologists usually measure the tumor length by using a ruler or by manually estimating tumor percentage. However, this is a time-consuming method, and the results may be inaccurate and vary depending on the pathologists. Despite conflicting findings about the superiority of measurement methods affecting clinical management, the use of DeepDx Prostate can provide various tumor quantitative data, such as tumor percentage and length, and these data can be useful for relating tumor volume to prognosis.

This study had several limitations. As there was no final diagnosis of IDC-P or high-grade prostatic intraepithelial neoplasia (HGPIN) solely without prostate adenocarcinoma in the validation set, accurate performance analysis of this lesion could not be performed, although the pathologist performed gland-level annotation for IDC-P, HGPIN, or ASAP lesions. In addition, we had insufficient data on precancerous lesions, such as IDC-P, HGPIN, and ASAP. We expected to improve the system performance to better identify these precancerous lesions by training more data including immunohistochemically stained slide images. We also planned to validate the system’s performance on these lesions by building path-level reference standards and performing path-level analysis.

In addition, we were not able to evaluate the system’s performance on data generated by different digital image scanners, which have different color tones in practice; in the future, we planned to evaluate the compatibility by training and evaluating our system using data scanned from different scanners.

Although our system clearly indicates the percentage of each Gleason pattern, validation was not possible because the pathologists were not asked to describe the percentage of each Gleason pattern, only tumor length. We also planned to investigate the relationship between grade groups and tumor volume, including the percentage of Gleason pattern, analyzed through our system with patients’ outcome by using survival data, including the recurrence rate. We would also try to find morphological phenotypes reflecting poor or better prognosis in a single grade group.

In addition, we would evaluate our system’s performance by comparing only pathologist-based diagnoses and diagnoses by a pathologist assisted by the proposed system.

## 4. Materials and Methods

### 4.1. Data Acquisition and Digitization

A total of 1,833 hematoxylin and eosin (H&E) stained glass slides of prostate needle biopsy cores along with their original hospital diagnoses were collected from two hospitals: the Korea University Guro Hospital, Seoul, Korea (Institutional Review Board Approval No. K2017-4488-001) and the Hanyang University Medical Center, Seoul, Korea (Institutional Review Board Approval No. 2018-10-010-002). We confirmed that all protocols in this study were performed in accordance with relevant regulations. The original glass slides from all cores included in the study were randomized and de-identified, and the institutional review boards of the two hospitals waived the need for informed consent from each patient. In this paper, we included cases of patients who underwent prostate core needle biopsy between 2010 and 2018.

After anonymizing patient data, the slides were digitized using Aperio AT2 scanners (Leica Biosystems Inc., Vista, CA, USA), at ×40 magnification, (i.e., resolution of 0.25 µm/pixel). After digitization, 700 images were selected for the validation set and balanced according to the grade groups reported in the original diagnosis. Specifically, we selected 100 acinar adenocarcinoma cases per grade group and 200 cases including 188 benign and 11 ASAP. However, owing to misdiagnosis later discovered in the original hospital diagnoses, one benign case was reclassified as grade group 3. The remaining 1133 cases were used for the discovery set, as detailed in Table 4.

### 4.2. Data Annotation

To develop the deep learning system, an experienced Korea board-certified pathologist provided gland-level accurate annotations for the 1133 WSIs in the discovery set by using annotation tools of DeepDx Prostate (Deep Bio Inc., Seoul, Korea). The pathologist blindly reviewed each core, marking tissue regions according to one of the following seven classes: benign tissue, acinar adenocarcinoma of Gleason patterns 3, 4, and 5, HGPIN, intraductal carcinoma of the prostate (IDC-P), and ASAP [4]. WSIs of high molecular weight cytokeratin (HMW-CK, Mouse monoclonal, Clone 34βE12, 1:200 dilution, Dako, Glostrup, Denmark) immunohistochemical stain of all cores except benign (according to original hospital diagnosis) were provided to the pathologist for accurate annotation.

To establish the reference standard diagnoses for the 700 WSIs in the validation set, three pathologists, who were not involved in the creation of the discovery set, reviewed each WSI independently for slide-level Gleason scores. The final Gleason scores were defined by majority vote, and in the case of a disagreement, the diagnoses of the genitourinary specialist were selected. Along with the Gleason scores, the pathologists were asked to identify each slide for the following characteristics: the necessity for immunohistochemistry, image quality in terms of slide preparation and digitization process, diagnostic difficulty of the case based on three levels (easy: cases whose diagnoses can be made simply by glancing at the WSIs; hard: cases that require consultation or immunohistochemical staining for diagnosis; medium: others), and tumor length if applicable. All the measurements, including Gleason scores, tumor lengths, and difficulty levels, were conducted using annotation tools of DeepDx Prostate.

To prevent any discrepancies that may arise from different color displays, the five pathologists were provided with identical hardware configurations (iMac Retina 5K display, 27 inches, model 2017; Apple Inc., Cupertino, CA, USA). Cases diagnosed as another carcinoma, such as urothelial carcinoma, were excluded from this study.

### 4.3. Automated Gleason Scoring System

The procedure of the proposed DeepDx Prostate system comprises two steps: patch-level segmentation and slide-level evaluation. In the first step, the target WSI is split into a grid of 704 × 704 pixel-sized image patches, which are then analyzed by the deep learning system in batches. The deep learning system retrieves a segmentation result per patch with respect to five categories: non-cancerous; Gleason patterns 3, 4, and 5; and IDC-P. In the second step, the patch results in a slide are aggregated into a single heatmap. The proportion of each Gleason pattern within the heatmap determines the final Gleason score (Figure S3 shows the overall algorithm workflow of our system). The deep learning system consists of two artificial neural networks of identical structure based on DeepLab v3+ [31] with additional non-local blocks [32] placed along with the connections between encoder and decoder of DeepLab v3+, extended from the original to accept image patches of the aforementioned size for the lesion segmentation. The networks were trained solely on the set of 940,875 image patches and the same number of corresponding region-level annotations, which was constructed by the patch extraction on a regular grid of size 352 × 352 from 1133 WSIs in the discovery set, followed by filtering out the patches with less than 10% being tissues. The networks were trained on eight-GPU machines using the stochastic gradient descent optimizer with Nesterov momentum. The initial learning rate was set to 0.01, which was multiplied by 0.1 for each 10 epochs during a total of 120 epochs. During training, we applied various random image transformations to augment the data. Specifically, image patches and corresponding annotations were rotated and flipped randomly, and then brightness, contrast, saturation, and hue were randomly modified to alleviate the effect of color variance from staining and scanning. In addition, image patches were extracted at sizes in the range of ±20%, and then resized to 704 pixels × 704 pixels to provide robustness against variation of magnification. The set of patches was randomly split into five nonoverlapping, equal-sized portions: A, B, C, D, and E. One network was trained using A, B, C, and D and tuned using E, while the other was trained using B, C, D, E, and tuned using A. The final system output was defined as the average of the two network outputs.

To further train the deep learning system, given the discovery set of limited size, we adopted hard-example mining after the initial network training, as in [25]. In the hard-example mining step, the system-generated patch segmentation results were evaluated using their mean intersection over union (mIoU) with the pathologist annotations at the beginning of each 15-epoch round. Patches with mIoU of less than 0.95 were considered as hard examples and selected for training. The snapshot and performance of each network on the tuning set were saved at each epoch. After 15 rounds of hard-example mining, the network snapshots associated with the highest performance were chosen for each network for the ensemble.

All the data preparation, network training, and evaluation were done with codes implemented in Python [33], using OpenCV [34] for tissue area detection by Otsu thresholding [35], and PyTorch [36] for the neural network implementation, training, and data augmentation.

### 4.4. Evaluation

The results from the validation set were evaluated by considering the grade groups corresponding to the Gleason scores generated by our system. We compared our system results to the established reference standard, individual diagnoses of each pathologist, and original diagnoses from the respective hospitals. The cases reported as ASAP by the original hospital diagnoses were analyzed separately.

The system performance was categorized according to the difficulty levels annotated by the pathologists, where easy, medium, and hard levels were labeled as 1, 2, and 3, respectively. The average difficulty level by the pathologists was rounded to the nearest integer to establish the reference standard of each case.

To assess the system performance based on the grade-group classification, Cohen’s kappa coefficient was used with and without quadratic weighting. For k number of classes, Cohen’s kappa coefficient κ is defined as following based on the number of elements in weight $w_{ij}$, in observed $x_{ij}$, and in expected matrices $m_{ij}$,

(1) $$\kappa =1-\frac{\sum_{i=1}^{k}\sum_{j=1}^{k}w_{ij}x_{ij}}{\sum_{i=1}^{k}\sum_{j=1}^{k}w_{ij}m_{ij}},$$

where κ is not weighted if $w_{ij}=0,\;1$ and is quadratically weighted if

(2) $$w_{ij}=\frac{{\left(i-j\right)}^{2}}{{\left(N-1\right)}^{2}}.$$

In addition to classification, we evaluated the system’s ability for quantifying cancer in terms of tumor length. Tumor length measurements from the original hospital diagnoses, those independently measured by pathologists using the annotation feature in DeepDx Prostate, and measurements generated by the system were compared. Pearson correlation coefficient R was used as the evaluation measure, and it is defined as

(3) $$R_{x,y}\;=\frac{Cov\left(X,\;Y\right)}{\sigma_{x}\sigma_{y}},$$

where X and Y are the compared random variables, σ represents the standard deviation of the corresponding variable, and *COV*() is the covariance. Only WSIs found to be Gleason 6 or above in all criteria (hospital diagnoses, evaluation by reference standard, and system-generated results) were considered in length comparisons. 

## 5. Conclusions

This paper proposed an automated Gleason scoring system based on deep neural networks for diagnosis of prostate core needle biopsy samples. The proposed system can assist pathologists to reduce the probability of over- or under-diagnosis by providing pathologist-level second opinions on the Gleason score when diagnosing prostate biopsies. We also expect that the proposed system will support research on prostate cancer treatment and prognosis by providing consistent and reproducible diagnosis.

## Figures and Tables

**Table 1:** Cohen’s kappa coefficient matrices for grade group classification (non-weighted/quadratic weighted).

	DeepDx Prostate	Reference Standard	Original Diagnoses	Pathologist 1	Pathologist 2	Pathologist 3
DeepDx Prostate	-	0.615/0.907	0.440/0.811	0.550/0.875	0.606/0.906	0.615/0.916
Reference standard	0.615/0.907	-	0.524/0.870	0.781/0.955	0.809/0.952	0.794/0.943
Original diagnoses	0.440/0.811	0.524/0.870	-	0.488/0.865	0.494/0.854	0.514/0.852
Pathologist 1 *	0.550/0.875	0.781/0.955	0.488/0.865	-	0.590/0.904	0.574/0.896
Pathologist 2	0.606/0.906	0.809/0.952	0.494/0.854	0.590/0.904	-	0.682/0.920
Pathologist 3	0.615/0.916	0.794/0.943	0.514/0.852	0.574/0.896	0.682/0.920	-

* Genitourinary specialist.

**Table 2:** Cohen’s kappa coefficient matrices according to diagnostic difficulty (non-weighted/quadratic weighted).

Difficulty		DeepDx Prostate	Reference Standard	Original Diagnoses
Easy	DeepDx Prostate	‒	0.656/0.958	0.634/0.853
	Reference standard	0.656/0.958	‒	0.611/0.836
	Original diagnoses	0.634/0.853	0.611/0.836	‒
Medium	DeepDx Prostate	‒	0.529/0.856	0.311/0.709
	Reference standard	0.529/0.856	‒	0.423/0.799
	Original diagnoses	0.311/0.709	0.423/0.799	‒
Hard	DeepDx Prostate	‒	0.224/0.525	0.255/0.508
	Reference standard	0.224/0.525	‒	0.224/0.683
	Original diagnoses	0.255/0.508	0.224/0.683	‒

**Table 3:** Cohen’s kappa score matrices according to diagnostic difficulty, between pathologists (non-weighted/quadratic weighted).

Difficulty		Pathologist 1 *	Pathologist 2	Pathologist 3
Easy	Pathologist 1 *	‒	0.931/0.990	1.000/1.000
	Pathologist 2	0.931/0.990	‒	0.931/0.990
	Pathologist 3	1.000/1.000	0.931/0.990	‒
Medium	Pathologist 1 *	‒	0.488/0.836	0.463/0.820
	Pathologist 2	0.488/0.836	‒	0.599/0.863
	Pathologist 3	0.463/0.820	0.599/0.863	‒
Hard	Pathologist 1 *	‒	0.273/0.636	0.382/0.815
	Pathologist 2	0.273/0.636	‒	0.219/0.733
	Pathologist 3	0.382/0.815	0.219/0.733	‒

* The genitourinary specialist.

**Table 4:** Number of slides in the entire dataset for this study.

Category	Discovery	Validation (Original Hospital Diagnosis)	Validation (Reference Standards)
Benign	645	188	203
ASAP *	0	11	8
Grade Group 1	145	100	67
Grade Group 2	131	100	111
Grade Group 3	27	101	92
Grade Group 4	122	100	61
Grade Group 5	63	100	158
Total	1133	700	700

* Atypical small acinar proliferation.

## Data Availability

The datasets used in this study are not publicly available at this moment due to data usage agreement restrictions. The de-identification process and usage of slides have been approved by the respective Institutional Review Boards (IRBs) of Korea University Guro Hospital and Hanyang University Medical Center. The source codes used in this paper is copyrighted by Deep Bio Inc. and is available only under appropriate license agreement with the copyright holder.

## Supplementary Materials

The following are available online at https://www.mdpi.com/2072-6694/11/12/1860/s1, Figure S1: Analysis result of poor quality images by DeepDx Prostate, Figure S2: Representative image of atypical small acinar proliferation case and high molecular weight cytokeratin (HMW-CK) staining, Figure S3: The overall algorithm workflow of our system, Table S1: Diagnoses for cases with atypical small acinar proliferation (ASAP) and respective immunohistochemical (IHC) staining requests.
