# Supplementary material for: Automated Gleason Scoring and Tumor Quantification in Prostate Core Needle Biopsy Images Using Deep Neural Networks and Its Comparison with Pathologist-Based Assessment
Source: Cancers (Basel). 2019 Nov 25;11(12):1860. doi: 10.3390/cancers11121860 (PMC6966453; doi:10.3390/cancers11121860)
Supplement: Supplementary file 1 [file cancers-11-01860-s001.pdf]

cancers

Supplementary Materials

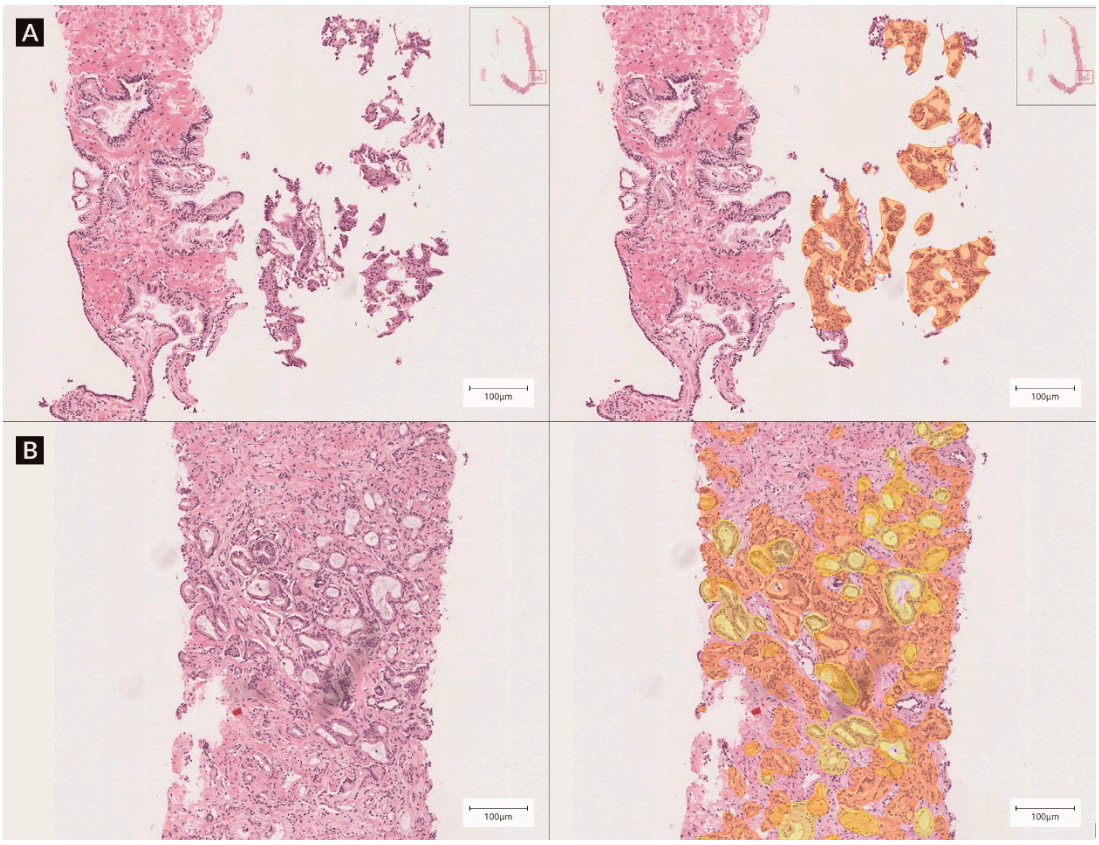

Figure S1. Analysis result of poor quality images by DeepDx Prostate. (**A**) Contains tissue fragments that have separated from the main body. (**B**) Contains a blurred area.

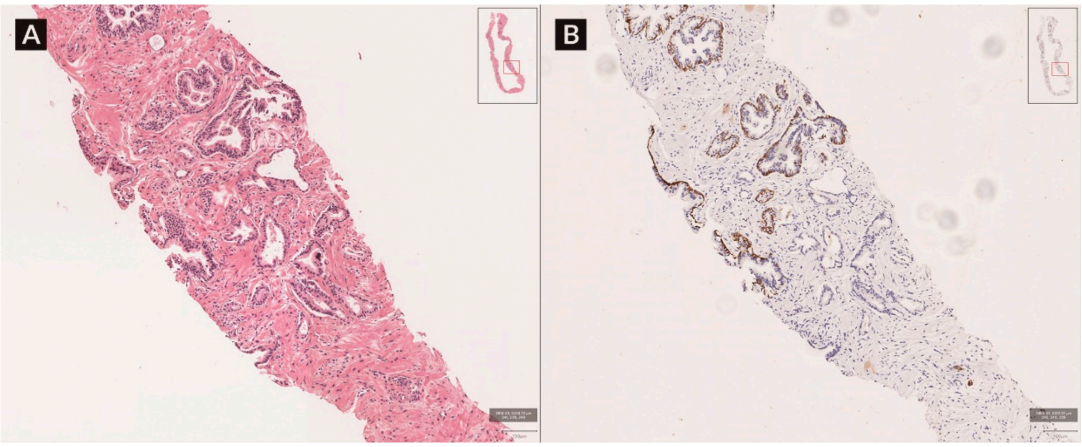

Figure S2. Representative image of atypical small acinar proliferation case and high molecular weight cytokeratin (HMW-CK) staining. Suspicious regions in (A) are confirmed as carcinoma in (B) through HMW-CK staining.

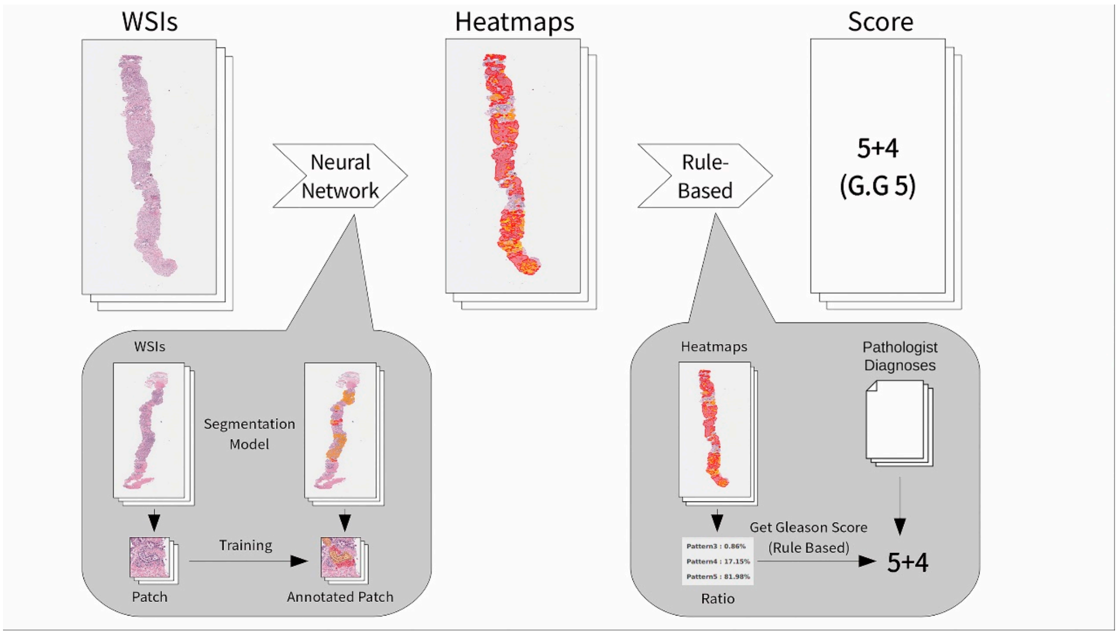

Figure S3. The overall algorithm workflow of our system.

Table S1. Diagnoses for cases with atypical small acinar proliferation (ASAP) and respective immunohistochemical (IHC) staining requests.

No.	Original diagnoses	Pathologist 1 *	Pathologist 2	Pathologist 3	DeepDx Prostate	IHC Requested
1	ASAP	benign	benign	ASAP	benign	yes
2	ASAP	group 1	group 1	ASAP	group 1	yes
3	ASAP	ASAP	benign	ASAP	benign	yes
4	ASAP	benign	ASAP	benign	benign	
5	ASAP	group 2	group 1	group 1	group 2	
6	ASAP	benign	benign	ASAP	benign	yes
7	ASAP	group 1	group 3	ASAP	group 3	yes
8	ASAP	benign	benign	ASAP	benign	yes
9	ASAP	group 1	group 1	group 1	group 2	
10	ASAP	benign	benign	benign	benign	
11	ASAP	group 1	group 1	group 1	group 2	

* The genitourinary specialist.
